# Reprogramming White Fat Cells for Adipose Manipulation Transplantation (AMT) Therapy

**DOI:** 10.21769/BioProtoc.5405

**Published:** 2025-08-05

**Authors:** Kelly An, Yusuke Ito, Nadav Ahituv

**Affiliations:** 1 Department of Bioengineering and Therapeutic Sciences, University of California San Francisco, San Francisco, CA, USA; 2 Institute for Human Genetics, University of California San Francisco, San Francisco, CA, USA; 3 Helen Diller Family Comprehensive Cancer Center, University of California San Francisco, San Francisco, CA, USA

**Keywords:** Adipocytes, Adipose organoids, CRISPR activation, Adipocyte reprogramming, Metabolic cancer therapy, Adipose manipulation transplantation (AMT)

## Abstract

Adipocytes are endocrine cells that function as the main energy storage in our body. They are commonly used in clinical procedures, including their removal via liposuction and transplantation in plastic surgery. Building on this, adipocytes can be used for ex vivo cellular manipulations, enabling therapeutic modifications that can provide beneficial clinical outcomes after transplantation. Here, we provide a detailed protocol on how to modify adipocytes and adipose organoids using CRISPR activation (CRISPRa), a technology termed adipose manipulation transplantation (AMT).

Key features

• This protocol is used to generate adipocytes and adipose organoids from human preadipocytes that can be genetically engineered for therapeutic purposes, including cancer metabolic therapy.

## Graphical overview


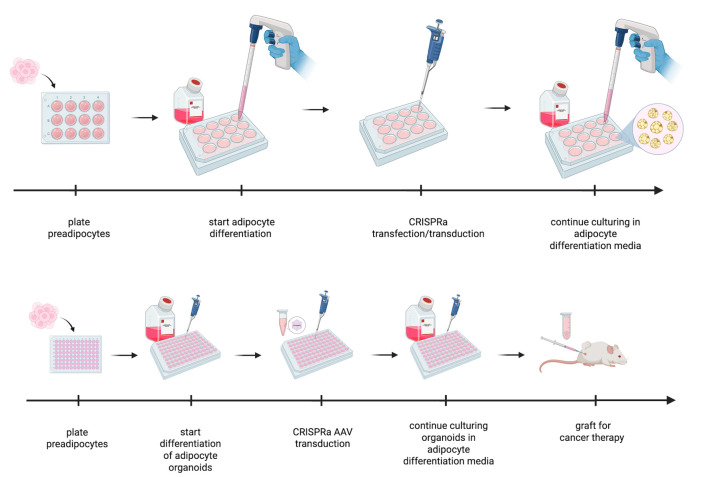


## Background

White adipose tissue (WAT) is a major energy storage organ and endocrine tissue. It primarily stores triglycerides and secretes adipokines, such as leptin, adiponectin, and other lipids that regulate various biological functions, including appetite, metabolism, and insulin homeostasis [1]. In contrast, brown adipose tissue (BAT) dissipates energy by non-shivering thermogenesis to maintain body temperature and was shown to be stimulated in adults upon cold exposure and to be inversely correlated with adiposity [2–6]. BAT not only has a high capacity for glucose and fatty acid (FA) uptake, but also secretes adipokines, all of which contribute to insulin sensitivity [7–9].

Subcutaneous WAT is capable of changing into a BAT-like tissue, called beiging [5,6]. Like brown adipocytes, beige adipocytes can convert energy to heat and contribute to whole-body energy expenditure. As ~90% of stored fat is subcutaneous in humans, inducing beiging by reprogramming WAT could be an effective strategy to improve metabolic outcomes. This is controlled by the upregulation of essential transcriptional regulators and enzymes, such as the uncoupling protein 1 (*UCP1*). In BAT, thermogenesis is mainly dependent on *UCP1* to “convert” substrates to heat; overexpression of this gene in mice leads to reduced WAT and body weight and increased recruitment of brown-like fat cells within WAT [10].

Liposuction and fat transplantation are commonly used in many surgical procedures, such as aesthetic and reconstructive surgery. Due to successful engraftment, adipose tissue transplantation could also be used for therapeutic treatments [11]. Several reports using rodent models have shown that BAT transplantation has beneficial metabolic outcomes [7,12–14]. In humans, there is a growing interest to carry out human adipose tissue grafting by using adipose organoids, as they have several advantages over WAT: 1) they can recapitulate the heterogeneity of tissue of origin, 2) they respond to endogenous stimuli, 3) they provide appropriate tissue microenvironments, and 4) they are more resistant to trauma and can provide long-term survival following transplantation [15–17].

Tumors are complex tissues composed of cancerous and non-cancerous cells in a hypoxic and nutrient-deprived microenvironment. The tumor microenvironment (TME) contains heterogeneous cell populations, including immune cells, mesenchymal support cells, and matrix components contributing to tumor growth and progression [18]. In order to survive this environment, tumors are capable of reprogramming metabolic pathways to better utilize available substrates in the surrounding TME [19]. During hypoxia, cancer cells also undergo metabolic reprogramming to increase lipid utilization [20,21]. Fatty acids (FAs) produce twice the energy of glucose. During periods of energy deprivation, WAT releases FAs into circulation by lipolysis. Cancer cells take up these FAs via the fatty acid transporter, CD36 [22]. Thus, WAT, the main source of circulating FAs, serves as an important energy source for cancer cells.

There are many efforts to target cancer glucose metabolism for therapeutic purposes (for a detailed review, see [19]). In addition, several drugs are also used to target lipid metabolism in cancer [23–26]. However, they have limited efficacy and off-target effects, and thus, very few of these drugs have made it into the clinic. CRISPR technology provides a novel way to modulate adipocytes and adipose stem cells to harness them for therapeutic purposes. We recently showed how these tools could be used to modify adipocytes or adipose organoids so that they suppress tumor progression [27], a technology termed adipose manipulation transplantation (AMT).

Here, we provide a detailed protocol on how to generate and manipulate adipocytes or adipose organoids for AMT using CRISPR activation (CRISPRa) with adeno-associated virus (AAV). We note that upregulation of genes could also be achieved via other modalities, such as zinc fingers, transcription activator-like effectors (TALEs), and cDNA expression vectors, and that other delivery methods could be used instead of AAV. In addition, while we mainly used *UCP1* as our target gene to generate “beige” cells or organoids [27], other genes could be targeted similarly. This includes not only BAT-associated genes but also genes involved in glucose transport, glucose metabolism, fatty acid oxidation, cholesterol synthesis, and many other metabolic pathways to outcompete cancer. We also note that these cells and organoids are great secretors and could be modified to secrete molecules that target the TME. Finally, this protocol could also be used to modify these cells and organoids, such that they can be used for other metabolic diseases. In summary, we provide a detailed experimental protocol for adipocyte and adipose organoid generation from human preadipocytes and how to reprogram them with CRISPRa. This technology holds promise not only for cancer therapy but also for many additional metabolic diseases.

## Materials and reagents

**Biological materials**

1. Human preadipocytes

2. Plasmid encoding guide RNA under the U6 promoter for the gene of interest; we used Addgene # 217015

3. Plasmid encoding a nuclease-deficient dCas9 fused with a transcriptional activator; we used Addgene # 115790

4. AAV9-encoding guide RNA under U6 promoter for gene of interest

5. AAV9-encoding dCas9 fused with a transcriptional activator

**Reagents**

1. DMEM, high glucose (Fisher Scientific, catalog number: 11-965-118)

2. Heat-inactivated FBS (Fisher Scientific, catalog number: 10438026)

3. Penicillin-streptomycin (Fisher Scientific, catalog number: 15140122)

4. Trypsin-EDTA 0.25% (Fisher Scientific, catalog number: 25200056)

5. DPBS, no calcium, no magnesium (Fisher, catalog number: 14190144)

6. Isobutyl-1-methylxanthine (IBMX) (Sigma-Aldrich, catalog number: 410957)

7. Dexamethasone (Sigma-Aldrich, catalog number: D1756)

8. Insulin (Sigma-Aldrich, catalog number: I9278)

9. X-tremeGENE^TM^ HP DNA transfection reagent (Roche, catalog number: 6366236001)

10. Opti-MEM^TM^ I reduced serum medium (Gibco, catalog number: 31985062)

11. Combimag^TM^ transfection reagent (Oz Biosciences, catalog number: CM21000)

12. DMEM, high glucose, pyruvate (Fisher Scientific, catalog number: 11-995-065)

13. *Trans*IT^®^-293 transfection reagent (Mirus, catalog number: MIR 2706)

14. AAVpro^®^ Purification kit maxi/midi (all serotypes) (Takara Bio, catalog number: 6666/6675)

15. AAVpro^®^ Titration kit (for real-time PCR) v. 2 (Takara Bio, catalog number: 6233)

16. KOD One^TM^ PCR master mix (Diagnocine, catalog number: TYB-KMM-201)

17. BstXI (Fisher Scientific, catalog number: FD1024); specific to this plasmid, but can be different for others

18. XhoI (Fisher Scientific, catalog number: FD0694); specific to this plasmid, but can be different for others

19. 10× FastDigest green buffer (Fisher Scientific, catalog number: FD1024)

20. QIAquick Gel Extraction kit (Qiagen, catalog number: 28704)

21. NEBuilder^®^ HiFi DNA Assembly master mix (NEB, catalog number: E2621)

22. SeaKem^®^ LE agarose (Lonza, catalog number: 50004)

23. Stellar^TM^ competent cells (Takara Bio, catalog number: 636766)

24. Luria Broth base (Miller’s LB broth base)^TM^, powder (Fisher, catalog number: 12795084)

24. LB agar plates with ampicillin, 100 mm (Teknova, catalog number: L1004)

25. Ampicillin, sodium salt powder (Corning, catalog number: 61-238-RM)

26. QiaPrep Spin MiniPrep (Qiagen, catalog number: 27104)

27. TRIzol reagent (Fisher Scientific, catalog number: 15596026)

28. Chloroform (Sigma-Aldrich, catalog number: 472476)

29. RNeasy Mini kit (Qiagen, catalog number: 74104)

30. qScript cDNA Synthesis kit (Quantabio, catalog number: 95047)

31. SsoFast EvaGreen Supermix (Bio-Rad, catalog number: 1725205)

32. Ultrapure DNase/RNase-free distilled water (nuclease-free water) (Fisher Scientific, catalog number: 10-977-023)

33. Clorox germicidal bleach (Clorox, catalog number: 30966)

**Solutions**

1. sgRNA PCR mix (see Recipes)

2. AAV backbone digestion mix (see Recipes)

3. NEBuilder^®^ HiFi DNA assembly reaction (see Recipes)

4. LB media (see Recipes)

5. Preadipocyte culturing medium (see Recipes)

6. Preadipocyte transfection mix (see Recipes)

7. Adipocyte differentiation medium (see Recipes)

8. Transfection mix (see Recipes)

9. Reverse transcription mix (see Recipes)

10. qPCR mix (see Recipes)

11. AAVproHEK293T culture medium (see Recipes)

12. AAV transfection mix (see Recipes)

13. AAV media change medium (see Recipes)

**Recipes**

**1. sgRNA PCR mix**

**Table:** 

Reagent	Final concentration	Quantity or Volume
10 μM forward primer	0.5 μM	1.5 μL
10 μM reverse primer	0.5 μM	1.5 μL
KOD One	1×	12.5 μL
Template (a sgRNA plasmid)	<250 ng	variable
Nuclease-free water		up to 25 μL
Total		25 μL

**2. AAV backbone digestion mix**

**Table:** 

Reagent	Quantity or Volume
AAV backbone	1 μg
BstXI	1 μL
XhoI	1 μL
10× FastDigest green buffer	5 μL
Nuclease-free water	42 μL
Total	50 μL

**3. NEBuilder^®^ HiFi DNA assembly reaction**

**Table:** 

Reagent	Quantity or Volume
Digested plasmid	50 ng
Insert	10 ng
NEBuilder^®^ HiFi DNA assembly master mix	10 μL
Nuclease-free water	up to 10 μL
Total	20 μL

**4. LB media**

**Table:** 

Reagent	Final concentration	Quantity or Volume
Luria Broth base	25 g/L	25 g
Distilled water		1 L
Total		1 L

**5. Preadipocyte culturing medium**

**Table:** 

Reagent	Final concentration	Quantity or Volume
DMEM, high glucose	89%	445 mL
Heat-inactivated FBS	10%	50 mL
Penicillin-streptomycin	1%	5 mL
Total		500 mL

**6. Preadipocyte transfection mix (per well of a 12-well plate)**

**Table:** 

Reagent	Quantity or Volume
Opti-MEM	100 μL
dCas9 plasmid	1 μg
gRNA plasmid	1 μg
X-tremeGENE reagent	2 μL
Total	~102 μL

**7. Adipocyte differentiation medium**

**Table:** 

Reagent	Final concentration	Quantity or Volume (for 10 mL)
DMEM, high glucose	90%	9 mL
Heat-inactivated FBS	10%	1 mL
IBMX	0.5 mM	10 μL (of 0.5 M stock)
Dexamethasone	1 μM	10 μL (of 1 mM stock)
Insulin	10 μg/mL	10 μL (of 10 mg/mL stock)
Total		~10 mL

**8. Transfection mix (per well of a 12-well plate)**

**Table:** 

Reagent	Quantity or Volume
Opti-MEM	100 μL
dCas9 plasmid	1 μg
gRNA plasmid	1 μg
X-tremeGENE reagent	2 μL
Combimag reagent	2 μL
Total	~104 μL

**9. Reverse transcription mix (per 1 sample)**

**Table:** 

Reagent	Volume
Nuclease-free water	22 μL
qScript reaction mix (5×)	8 μL
qScript RT	2 μL
Total	32 μL

**10. qPCR mix (per 1 sample)**

**Table:** 

Reagent	Volume
SsoFast EvaGreen supermix	5 μL
Forward primer (final concentration 300–500 nM)	0.25 μL
Reverse primer (final concentration 300–500 nM)	0.25 μL
Nuclease-free water	1.5 μL
Total	7 μL

**11. AAVproHEK293T culture medium**

**Table:** 

Reagent	Final concentration	Quantity or Volume
DMEM, high glucose, pyruvate	89%	445 mL
Heat-inactivated FBS	10%	50 mL
Penicillin-streptomycin	1%	5 mL
Total		500 mL

**12. AAV transfection mix (per one 150 mm dish)**

**Table:** 

Reagent	Quantity or Volume
Opti-MEM	4 mL
*Trans*IT^®^-293 transfection reagent	120 μL
Your own vector (either gRNA or dCas9-VP64)	20 μg
pHelper vector	20 μg
pRC9 (AA9 serotype) vector	20 μg
Total	

**13. AAV media change medium**

**Table:** 

Reagent	Final concentration	Quantity or Volume
DMEM, high glucose, pyruvate	98%	490 mL
Heat-inactivated FBS	2%	10 mL
Total		500 mL

**Laboratory supplies**

1. Nunclon Sphera-treated 96-well U-shaped-bottom microplate (Thermo Scientific, catalog number: 174925) (for adipose organoids)

2. Common cell culture supplies (cell culture plates/flasks/consumables, pipettes, pipet-aid, serological tips)

3. Common laboratory consumables (gloves, pipettes, pipette tips, centrifuge tubes)

## Equipment

1. Water/bead bath (Thermo Fisher Scientific, model: TSGP05)

2. Tissue culture incubator (37 °C, 5% CO_2_) (Thermo Fisher Scientific, model: 3110)

3. Tissue culture hood (Thermo Fisher Scientific, model: 1300 Series A2 Biosafety Cabinet)

4. Magnetic plate for magnetofection (Oz Biosciences, model: MF10000)

5. Centrifuge with refrigeration that can spin 15 or 50 mL conical tubes (Beckman Coulter, model: B08708)

6. Microcentrifuge with refrigeration capability (Eppendorf, model: 5417R)

7. Vortex mixer (Fisher Scientific, model: Vortex-Genie 2 Mixer)

8. Heat block (Fisher Scientific, model: 88-870-001)

9. PCR machine (ProFlex PCR System, Fisher Scientific, model: 4484073)

10. Bacterial shaking incubator (New Brunswick, model: Excella E24)

11. Plate mixer (Fisher Scientific, model: 88-861-023)

12. NanoDrop 8000 (Fisher Scientific, model: ND-8000-GL)

13. qPCR machine (Thermo Fisher Scientific, model: QuantStudio 6)

## Procedure

**A. Optimizing CRISPR**

1. To target promoters, use CRISPick [28] or other sgRNA design tool for CRISPRa, select your reference genome and type of Cas enzyme, enter the target gene name, and pick the top 5 sequences. For enhancers, you will need to upload the sequence you want to target.

2. Order primers with flanking restriction enzyme sites and prepare 100 μM stock solutions using dH_2_O.

a. sasgRNA_F-Inf-*BstX*I: gagaaCCAcctTGTTGG(your sgRNA-seq)gttAtagtactctgg

b. sasgRNA_RevInf*Xho*I (universal): ggatccTAGTActcgagAAaaaaatctc

3. Prepare sgRNA PCR mix (see Recipes) to synthesize the DNA fragment with PCR using gRNA vector as the template with the primers. Specific temperature will depend on the chosen primers, but see Table 1 as an example.

**Table 1.** PCR setup

Step	Temperature	Time
Initial denaturation	98 °C	1 min
Cycling 32 cycles	98 °C	10 s
	60 °C	5 s
	68 °C	2 s
Final extension	68 °C	1 min (15–30 s per kb)

4. Measure DNA concentration.

5. Prepare AAV backbone digestion mix (see Recipes) for 1–3 h of AAV backbone digestion using BstXI and XhoI.

6. Make a 1% agarose gel.

7. Run inserts and the digested vector on the 1% agarose gel. Visualize the gel and then excise the bands for the digested vector and sgRNA.

8. Perform DNA extraction on the bands using the QIAquick Gel Extraction kit.

9. On ice, set up NEBuilder^®^ HiFi DNA assembly reaction (see Recipes).

10. Incubate samples in a thermocycler at 50 °C for 15 min, if 2 or 3 fragments are being assembled, or for 60 min, if 4–6 fragments are being assembled.

11. Transform Stellar competent cells with 2 μL of the chilled assembled product following Takara’s Stellar™ Competent Cells protocol PT5055-2.

12. Pick colonies and inoculate overnight.

a. Make LB medium containing ampicillin (1:500) and add 3 mL per 14 mL polyethylene round tube.

b. Pick one colony from the LB-ampicillin culture plate from your transformation step and add to each tube.

c. Incubate in a bacterial shaking incubator at 37 °C with shaking at 250× g for 7–8 h.

d. Continue to miniprep.

13. Perform a plasmid miniprep with the QiaPrep Spin MiniPrep.

14. Send for sequencing to validate proper cloning of sgRNA.

15. Store your plasmids at -20 °C.

**B. CRISPRa optimization via transfection in preadipocytes**

**B1. Preadipocyte culture**

1. Make preadipocyte culturing medium (see Recipes). Prewarm media and trypsin-EDTA to 37 °C.

2. Thaw cells and maintain preadipocytes below 80% confluency. <85% confluency can impact differentiation.

3. Culture these cells with preadipocyte culturing medium. Change media every 2–3 days or when the media turns yellow, and keep cells incubated in a tissue culture incubator with 5% CO_2_ at 37 °C.

**B2. Transient transfection**

1. Plate a total of 5 × 10^5^ human preadipocytes onto 12-well plates.

2. Check the plated cells to make sure they are at 100% confluency.

3. Replace preadipocyte culturing media (see Recipes) and incubate for 48 h in a tissue culture incubator with 5% CO_2_ at 37 °C.

4. Prepare adipocyte differentiation media (see Recipes). Warm the media to 37 °C.

5. Aspirate media from preadipocyte culture.

6. Add adipocyte differentiation media.

7. Place the plate back into the incubator.

8. Change media every other day. On day 4 of differentiation, start thawing X-tremeGENE HP transfection reagent 30 min before transfection.

10. Prepare an adequate amount of preadipocyte transfection mix for the number of wells you are working with (see Recipes).

11. Incubate transfection mix for 15 min at room temperature.

12. Add the appropriate amount of the mixture dropwise into each well.

13. Return the plate to the 37 °C incubator.

14. Change the media the next day.

**B3. RNA extraction and cDNA synthesis**

1. Discard media from the culture at day 8 of differentiation.

2. Wash cells with DPBS.

3. Add 500 μL per well of TRIzol and shake the 12-well plates with a plate mixer for 5 min.

4. Collect cell lysate from the wells into 1.5 mL tubes.

5. Add 100 μL of chloroform and mix well with a vortex.

6. Centrifuge at 20,000× *g* for 10 min at 4 °C.

7. Transfer the supernatant into new 1.5 mL tubes filled with 150 μL of 70% ethanol.

8. Transfer the mixture into a spin column from the Qiagen RNeasy kit.

9. Spin down the column to discard the flowthrough.

10. Wash the spin column with 700 μL of buffer RW1.

11. Spin down the column to discard the flowthrough.

12. Wash the spin column with 500 μL of buffer RPE.

13. Spin down the column to discard the flowthrough.

14. Repeat steps B3.11–13 again.

15. Set the column to a new 1.5 mL tube.

16. Add 50 μL of nuclease-free water to the column.

17. Centrifuge at 10,000× *g* for 1 min at room temperature.

18. Collect the RNA solution in the 1.5 mL tube.

19. Measure total RNA concentration with NanoDrop 8000 or a compatible spectrophotometer.

20. Dilute RNA with nuclease-free water to equalize the RNA concentration among samples.

21. Mix 8 μL of the diluted RNA with reverse transcription mix (see Recipes) in a 96-well plate and run reverse transcription with the PCR machine (Table 2).

**Table 2.** Reverse transcription setup

Step	Temperature	Time
1 cycle	22 °C	5 min
1 cycle	42 °C	30 min
1 cycle	85 °C	5 min
Storage	4 °C	hold

**B4. qPCR**

1. Dilute the reverse-transcribed cDNA solution between 5 and 10-fold, as appropriate for your downstream application, with nuclease-free water.

2. Mix the 3 μL diluted cDNA solution with qPCR mix (see Recipes) in 96- or 384-well plates for qPCR and run qPCR with the qPCR machine (Table 3).

**Table 3.** qPCR setup

Step	Temperature	Time
Initial denaturation	95 °C	20 s
Cycling 40 cycles	95 °C	1 s
	60 °C	20 s
Melting curve analysis	65 to 95 °C	2–5 s/step

3. Calculate gene expression level according to the ΔΔCt method.

a. Calculate the difference in Ct values (ΔCt) between the gene of interest and the housekeeping gene.

b. Calculate the difference between the ΔCt of the experimental samples and the ΔCt of the control sample.

4. Select sgRNA according to gene expression induction.

**C. AAV generation of dCas9-activator and sgRNA**

**C1. AAV production**

1. Culture low-passage-number AAVproHEK293T cells.

2. Plate 2.5 million AAVproHEK293T cells per 150 mm dish in 30 mL of AAVproHEK293T culture medium (see Recipes).

3. Incubate for 24 h or until they reach 80%–90% confluency.

4. Prepare an adequate amount of AAV transfection mix for the number of plates you are working with (see Recipes). Gently invert to mix and incubate for 15 min at room temperature before proceeding with transfection.

5. Add AAV transfection mix (approximately 4 mL) dropwise to each 150 mm plate.

6. Incubate the 150 mm plate(s) at room temperature for 8 min and check that the solution appears foggy.

7. Place plate(s) in a 37 °C incubator and incubate for 24 h.

8. Prepare AAV media change medium (see Recipes) and warm to 37 °C.

9. Aspirate media from each 150 mm plate.

10. Replenish with AAV media change medium carefully by tilting the plate slightly and gently adding media to the edge of the plate. Avoid directly pipetting onto the cells to prevent them from detaching.

11. Return the plate to the 37 °C incubator and incubate for 3–7 days before proceeding to AAV purification.

**C2. AAV purification, day 1**

1. Cool down centrifuges to 4 °C and prepare 10% bleach for AAV waste.

2. Add 1 volume of 0.5 M EDTA (pH 8.0) per 80 volumes of culture medium to detach cells. Gently swirl to mix.

3. Incubate at room temperature for 5–10 min for cells to detach.

4. Use a pipette to take up media and rinse up and down the plate surface to wash down the remaining cells.

5. Collect cells into a 50 mL conical tube.

6. Centrifuge at 1,800× *g* for 10 min at 4 °C.

7. Discard the supernatant.

8. Centrifuge again at 1,800× *g* for 1 min at 4 °C. Make sure to completely remove the remaining supernatant. Leftover media can change pH and impact virus concentration.

9. Loosen the cell pellet well by vortexing for approximately 5 s, which should result in a sauce-like consistency without clumps.

10. Add 1 mL of AAV extraction solution A (Takara AAVpro^®^ Purification kit) per one 150 mm dish.

11. Resuspend by vortexing for 15 s.

12. Incubate for 10 min at room temperature.

13. Vortex again for 15 s.

14. Transfer the solution to sterile 2 mL centrifuge tubes with 1 mL per tube.

15. Centrifuge at 4,000× *g* for 10 min at 4 °C.

a. Repeating steps C2.10–15 may increase the yield. Centrifuge at 8,000× *g* the second time.

16. Transfer the supernatant to new sterile 2 mL tubes.

17. Add 1 volume of AAV extraction solution B (Takara AAVpro^®^ Purification kit) per 10 volumes of extraction A to the supernatant. In this case, it would be 100 μL of extraction solution B.

18. Store virus suspension at -80 °C or proceed directly to day 2 of AAV purification and concentration.

**C3. AAV purification and concentration**

1. Cool centrifuge (for 15 mL/50 mL conical) to 15 °C and the microcentrifuge to 4 °C.

2. Preheat a heat block for 2 mL tubes to 37 °C.

3. If the virus suspension was stored at -80 °C, thaw at 37 °C for 5–10 min.

4. Prepare 10% bleach for AAV waste.

5. Add 1 volume of cryonase cold-active nuclease (Takara AAVpro^®^ Purification kit) (11 μL) per 100 volumes of the virus suspension (1,100 μL).

6. Incubate for 1–2 h at 37 °C.

7. Add 1 volume of precipitator A (Takara AAVpro^®^ Purification kit) (111.1 µL) per 10 existing volumes (1,111 µL) and immediately vortex for 10 s.

8. Incubate at 37 °C for 30 min, then vortex again for 10 s.

9. Add 1 volume of precipitator B (Takara AAVpro^®^ Purification kit) (61.1 μL) per 20 existing volumes (1222.1 μL) and immediately vortex for 10 s.

10. Centrifuge at 9,000× *g* for 5 min at 4 °C.

11. Prepare tubes to pool the supernatant containing the same virus from the 2 mL tubes together.

a. For the Midi Prep kit, 1× 150 mm dish can be processed per 15 mL of Amicon Ultra-4, 100 kDa filter column with the clear Millex-HV 0.45 μm filter.

b. For the Maxi Prep kit, 10 × 150 mm dishes can be processed per 50 mL of Amicon Ultra-5, 100 kDa filter column with the Millex-HV 0.45 μm filter with a yellow rim.

12. Filter the supernatant using the corresponding Millex-HV 0.45 μm filter into the Amicon Ultra column.

a. Prepare 1 × 100 Soft-Ject syringes.

b. Place the Amicon Ultra column in a tube rack. Pull out the plunger of the syringe and twist the tip of the syringe onto the filter. Put the connected syringe filter on top of the open column. Pour supernatant into the syringe. Reinsert the plunger and then push down in one smooth motion. Stop when you encounter resistance and do not push past the breaking point of the filter or pull back. Hold the plunger in place to get rid of the remaining filtrate.

13. Discard the syringe and filter into 10% bleach, cap the column tube, and centrifuge at 2,000× *g* for 5 min at 15 °C.

14. Confirm that the AAV solution in the filter device is <0.4 mL. If the volume of the solution is >0.4 mL, repeat the centrifugation.

15. Discard the flowthrough and add 1 mL per initial 150 mm plate of suspension buffer in the Amicon Ultra

column.

16. Centrifuge at 2,000× *g* for 5 min at 15 °C. Repeat centrifugation if the volume is >0.4 mL after the spin.

17. Repeat steps C3.15–16 four more times for a total of five washes.

18. Discard the flowthrough. Transfer the virus suspension in the column to a new 1.5 mL tube.

19. Aliquot 2 μL for AAV titration. Store the virus at -80 °C.

20. Use the AAVpro^®^ Titration kit (for real-time PCR) v. 2 for AAV titration.

**D. Culturing preadipocytes for differentiation**

1. Make preadipocyte culturing medium (see Recipes). Prewarm media and trypsin-EDTA to 37 °C.

2. Thaw cells and maintain preadipocytes below 80% confluency. Confluency higher than 85% can impact differentiation. Culture these cells with preadipocyte culturing medium.

3. When ready to seed cells for differentiation:

a. Wash cells with DPBS.

b. Use trypsin-EDTA and incubate in an incubator for 5 min to dissociate.

c. Use preadipocyte culturing medium to neutralize trypsin. Collect cells in solution into a conical tube.

d. Spin down cells in a centrifuge at 300× *g* for 5 min.

e. Aspirate supernatant and resuspend the cell solution in 1 mL of preadipocyte culturing medium.

f. Count cells.

4. Seed cells for adipocyte differentiation:

a. Use Thermo Fisher Scientific Useful Cell Numbers for Cell Culture to seed at confluency for differentiation. For example, seed 5 × 10^5^ cells per well of a 12-well plate.

b. If seeding for adipocyte organoid formation, seed 1.5–5 × 10^5^ cells per well with 100 μL per well in the Nunclon Sphera plate. Twenty-four to forty-eight hours after seeding, proceed to Section F2.

**E1. Differentiating human preadipocytes to adipocytes**

1. Check plated cells to make sure they are at 100% confluency.

2. Replace preadipocyte culturing media (see Recipes) and incubate for 48 h.

3. Prepare adipocyte differentiation media (see Recipes). Warm the media to 37 °C.

4. Aspirate media from preadipocyte culture.

5. Add adipocyte differentiation media.

6. Place the plate back into the incubator.

7. Change media every other day for 14–21 days. Change media using a pipette; do not use a vacuum aspirator, as it can disrupt adipocytes, which are more fragile due to the lipid droplets that will form.

a. Adipocytes will start to form lipid droplets that look like clusters of small, empty/clear circular structures in the cell.

**E2. Differentiating human preadipocytes to adipose organoids**

1. Twenty-four to forty-eight hours after seeding human preadipocytes in a Nunclon Sphera plate, prepare adipocyte differentiation media (see Recipes) and warm to 37 °C.

2. Observe that the cells have formed spherical organoid structures at the bottom of the wells. Using a pipette, carefully remove the preadipocyte growth media. Work with caution so that you do not suck up the organoids.

3. Add 100 µL of adipocyte differentiation media to each well.

4. Change media every day for 21–28 days by pipetting. Pipette out half of the media and add 50 μL of new differentiation media.

a. Be mindful that evaporation can occur, so removing half of the media can be less than 50 μL.

b. Be cautious of sucking up adipose organoids, which are small and in suspension.

**F. CRISPRa browning of human adipocytes or adipose organoids**

**F1. Transfecting adipocytes**

1. On day 4 of adipocyte differentiation, change the cell media (adipocyte differentiation media).

2. Prepare an adequate amount of CRISPRa transfection mix for the number of wells you are working with (see Recipes).

3. Incubate transfection mix for 15 min at room temperature.

4. Add the appropriate amount of the mixture dropwise into each well.

5. Place the culture plate on the magnetofection magnetic plate for 30 min.

6. Return the plate to the 37 °C incubator.

7. Change media 6 h after transfection or the next day.

**F2. AAV transduction**

1. For adipocytes, after day 2 of adipocyte differentiation, add AAVs with an MOI of 1 × 10^6^.

2. For adipose organoids, if grafting for cancer therapy: One week before grafting adipose organoids, treat organoids with AAVs with an MOI of 1 × 10^6^.

## Validation of protocol

All validation data and information about the analysis are included in the original research article:

- Nguyen et al. [27] Implantation of engineered adipocytes suppresses tumor progression in cancer models. *Nature Biotechnology*.

The original research article confirms browning activation in white adipocytes after CRISPR activation (Extended Data Figure 1, Nguyen et al. [27]). It also shows that oxygen consumption rates, glucose uptake, and fatty acid oxidation are increased in these CRISPRa-modulated adipocytes (Extended Data Figure 1, Nguyen et al. [27]).

Additionally, the protocol to reprogram adipocytes is shown in [27] to be an effective method of metabolic therapy to target cancer. Cancer cell growth is shown to be suppressed when co-cultured with the CRISPRa reprogrammed human adipocytes (Figure 1, Nguyen et al. [27]). Glucose uptake of cancer cells is also reduced when these cells are co-cultured with CRISPRa-modulated human adipocytes (Figure 1, Nguyen et al. [27]). Cancer cells also exhibit decreased glucose utilization, as shown by measurements of the extracellular acidification rate (ECAR) and metabolic gene expression (Figure 1, Nguyen et al. [27]).

Using this protocol, *UCP1*-CRISPRa-engineered adipose organoids were generated and co-transplanted in mice to suppress the growth of xenografted tumors (Figure 2, Nguyen et al. [27]).

## General notes and troubleshooting

**General notes**

1. Differentiation of adipocytes will take 14–21 days; differentiation of adipose organoids will take 21–28 days and requires media changes every other day.

**Troubleshooting**

Problem 1: Low AAV concentration.

Possible causes: Low transfection efficiency or ineffective purification.

Solution: **Cells should be at about 70%–80% confluency when starting transfection.** Waiting 72 h or longer, up to 7 days, after the media change (step C1.11) can help improve concentration. Repeating steps C2.10–15 and centrifuging at 8,000× *g* the second time can help increase the yield by about 10%.

Problem 2: Not seeing lipid droplets forming.

Possible cause: Disruption of lipid droplets or not allowing preadipocytes to reach confluency before starting differentiation.

Solution: **Make sure preadipocytes have reached 90%–100% confluency before beginning differentiation.** Take care during media changes to not disturb the cells by aspirating at the edge of the well using a manual pipetting method. Leave some media behind to avoid completely drying out the cells and replenish with new media in a gentle manner. Tilt the plate at an angle and dispense media at the edge of the well to avoid disrupting cells. Continue to change media for longer than 14 or 21 days to ensure lipid droplets are formed, indicating efficient differentiation.
